# Pyomyosite tuberculeuse primitive chez une patiente immunocompétente: à propos d'un cas

**DOI:** 10.11604/pamj.2014.19.200.3099

**Published:** 2014-10-24

**Authors:** Alifa Daghfous, Khaled Bouzaidi, Lamia Rezgui Marhoul

**Affiliations:** 1 Service d'Imagerie Médicale, Centre Traumatologie et des Grands Brûlés, Tunis, Tunisie; 2 Service d'Imagerie Médicale, Hôpital MT Maâmouri, Nabeul

**Keywords:** Tuberculose, pyomyosite, imagerie par résonance magnétique, Tuberculosis, pyomyositis, MRI

## Abstract

L'atteinte du muscle strié au cours de la tuberculose chez un sujet immunodéprimé ou à partir de l'extension d'un foyer ostéo-articulaire de voisinage est rare mais bien connue. Toutefois, l'atteinte primitive est exceptionnelle ainsi que celle chez un sujet immunocompétent. L'imagerie par résonnance magnétique (IRM) est d'un grand apport pour le diagnostic avec une excellente sensibilité. Nous rapportons un cas de pyomyosite tuberculeuse primitive du mollet chez une patiente non immunodéprimé tout en précisant l'apport de l'IRM.

## Introduction

La pyomyosite tuberculeuse représente une localisation exceptionnelle de la tuberculose. Il s'agit d'une suppuration du muscle strié d’évolution chronique et insidieuse, plus fréquente chez le sujet immunodéprimé [1]. Elle est secondaire le plus souvent à une extension à partir d'un foyer de voisinage ostéo-articulaire ou tendineux. Dans des cas extrêmement rares, elle est primitive et survient même en l'absence de facteurs de risque. Nous rapportons l'observation d'une pyomyosite tuberculeuse primitive du mollet gauche chez une patiente immunocompétente tout en précisant l'apport de l'imagerie par résonance magnétique (IRM).

## Patient et observation

**Figure 1:**
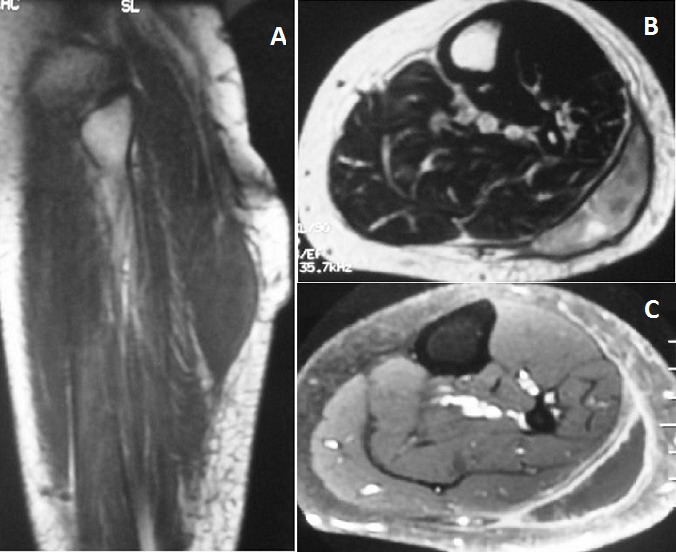
IRM de la jambe gauche (coupe coronale pondérée T1 (a), axiale pondérée T2 avec suppression du signal de la graisse (b), axiale T1 après injection de gadolinium et suppression du signal de la graisse (c)). Collection liquidienne de la loge postéro externe du mollet en hyposugnal T1 (a), hypersignal hétérogène T2 (b) et limitée par une coque épaisse prenant le contraste (c)

Mme J S, âgée de 46 ans, sans antécédent pathologique, a consulté pour une tuméfaction douloureuse du mollet gauche apparue il y a un an, ayant augmenté progressivement de taille. Aucune notion de traumatisme ni de plaie n'a été rapportée. L'examen physique a trouvé une tuméfaction douloureuse de la face postéro-latérale du mollet gauche, de consistance molle, de 10 cm de grand axe et sans signes inflammatoires locaux en regard chez une patiente en bon état général, apyrétique. Le reste de l'examen somatique était sans particularité. A la biologie, l'hémogramme était normal. Il existait un syndrome inflammatoire biologique avec une vitesse de sédimentation à 60 mm à la première heure et une C-réactive protéine à 40,6 mg/l. La radiographie de la jambe gauche était sans anomalie. Une échographie du mollet gauche a objectivé une masse de la loge postéro-externe de 10cm de grand axe d'aspect non spécifique. L'IRM a mis en évidence une masse fusiforme du gastrocnémien latéral à centre liquidien, en hyposignal T1, hypersignal hétérogène T2, limitée par une coque périphérique rehaussée après injection de gadolinium (Figure 1). Cette masse ne comportait pas une composante hémorragique. Les deux os de la jambe étaient de signal et de rehaussement normaux. Devant cet aspect IRM, une lésion tumorale kystisée a été évoquée et un traitement chirurgical a été indiqué. La tomodensitométrie (TDM) thoraco-abdominopelvienne réalisée dans le cadre d'un bilan d'extension était sans anomalie. Les données per-opératoires confirmaient la nature liquidienne de la masse avec issu de liquide dont l'aspect faisait évoquer du caséum. L'examen anatomopathologique définitif a conclu à une tuberculose caséo-folliculaire du mollet. Une quadruple antibiothérapie anti-tuberculeuse a été prescrite pendant 2 mois, relayée par une bithérapie pendant 8 mois. L’évolution clinique et radiologique était favorable avec régression totale de la collection à l'IRM de contrôle faite à 12 mois de traitement.

## Discussion

Les pyomyosites tuberculeuses sont des suppurations du muscle strié d’évolution chronique et insidieuse rarement rapportées. Elles surviennent chez 2% des sujets atteints de tuberculose et/ou immunodéprimés [2]. Dans des cas extrêmement rares, cette atteinte est primitive. Dans une étude rétrospective faite par Soler et al [3], l'incidence de la pyomyosite tuberculeuse primitive a été estimée à 0,0026%. Petter [4] a trouvé également dans son étude une incidence faible estimée à 0,015%. Dans les zones endémiques, l'incidence est beaucoup plus élevée où elle a été évaluée à 1,8% [2, 5]. L’étiopathogénie de la localisation musculaire primitive du *Mycobactérium tuberculosis* est encore mal connue. Elle peut être due à une fragilisation du muscle par une affection pré-existante tels que les traumatismes, la chirurgie, la présence de corps étrangers, l'ischémie, les connectivites et l'immunodépression [6–8]. Pour notre patiente, aucune cause n'a été retrouvée. Tous les muscles du squelette peuvent être touchés avec une atteinte prédominante de ceux de la cuisse [7]. L’âge de survenue est généralement avancé, entre 50 et 70 ans [7]. Le diagnostic clinique de la pyomyosite reste difficile, en raison d'une symptomatologie clinique non spécifique surtout à un stade précoce [9]. De ce fait, l'imagerie est d'un grand apport dans l'approche diagnostique. L’échographie et la TDM restent moins performantes que l'IRM. L’échographie peut montrer une infiltration des fibres musculaires aux stades précoces ou un abcès déjà constitué [10]. La TDM peut mettre en évidence un élargissement musculaire et un effacement des plans graisseux intramusculaires. L'IRM est considérée comme la technique de choix car elle permet d'orienter le diagnostic avec une excellente sensibilité [11]. A un stade précoce, elle montre l'inflammation du muscle sous la forme d'une hyper-intensité mieux visible en séquence pondérée T2 ou en séquence T2 avec saturation du signal de la graisse. Au stade d'abcès, l'IRM met en évidence une collection de signal liquidien entourée d'une coque plus ou moins épaisse rehaussée après injection de gadolinium [11, 12]. Elle permet également de confirmer le caractère primitif de la pyomyosite et d'apprécier l'extension et les rapports avec les structures de voisinage [3] afin de planifier la conduite thérapeutique.

Les techniques d'IRM avancées telles que les séquences de diffusion et la spectroscopie sont d'un grand apport pour le diagnostic de la pyomyosite tuberculeuse [13]. En effet, l'imagerie de diffusion avec le calcul du coefficient de diffusion apparent (ADC) permet d'orienter vers le diagnostic de pyomyosite en montrant un ADC qui est très bas et de s'affranchir ainsi aux diagnostics différentiels tels que les tumeurs nécrosées qui sont en hyposignal sur les séquences de diffusion avec un ADC qui est élevé. La spectroscopie permet d’évoquer l'origine tuberculeuse devant l’élévation du pic lipide/lactate. Le diagnostic de certitude de la tuberculose reste bactériologique avec identification de la mycobactérie [14]. Le traitement est médico-chirurgical associant le traitement antituberculeux classique et le drainage chirurgical de l'abcès [7].

## Conclusion

La pyomyosite tuberculeuse primitive représente une localisation exceptionnelle de la tuberculose. Elle doit être évoquée même chez le sujet immunocompétent en particulier en zone d'endémie. L'IRM avec les séquences classiques et les techniques avancées (séquences de diffusion et spectroscopie) est d'un grand apport dans l'approche diagnostique, la conduite à tenir thérapeutique et le suivi évolutif.
